# A comparison of clinicopathologic features of prostate cancer between Nigerian and South African black men

**DOI:** 10.1186/s12301-022-00273-y

**Published:** 2022-03-05

**Authors:** Ridwan Oladotun Ahmed, Vikash Sewram, Adisa Rasaaq Oyesegun, Birhanu Ayele, Abrie van Wyk, Pedro Fernandez

**Affiliations:** 1 African Cancer Institute, Department of Global Health, Stellenbosch University, PO Box 241, Tygerberg, 8000 South Africa; 2 Department of Radiotherapy and Oncology, National Hospital, Plot 132, Central District (Phase II), P.M.B 425, Garki, Abuja, Nigeria; 3 Mount Vernon Cancer Centre, Rickmansworth Road, Northwood, HA6 2RN UK; 4 Division of Anatomical Pathology, Department of Pathology, Stellenbosch University, PO Box 241, Cape Town, 8000 South Africa; 5 Division of Urology, Department of Surgical Sciences, Stellenbosch University, PO Box 241, Cape Town, 8000 South Africa

**Keywords:** Prostate cancer, PSA, Gleason score, Black men, African ancestry, Age, Risk factors

## Abstract

**Background:**

Globally, prostate cancer (PCa) is the commonest non-cutaneous male malignancy. It is more aggressive among black men with little known reasons as to the cause and continued trend among black men. This disproportionate pattern of PCa especially among black men of African ancestry resident in Africa calls for a closer look. Nigeria and South Africa, combined, have the highest cumulative risk incidence of PCa in Africa. The present study investigated the clinicopathologic behaviour of PCa among Nigerian and South African black men and the relationship between the disease and socio-demographic characteristics alongside medical co-morbidities.

**Methods:**

A retrospective cross-sectional study was undertaken in which de-identified records of 234 black men with pathologically confirmed PCa between 2007 and 2017 from two tertiary hospitals, in Nigeria (National Hospital, Abuja) and South Africa (Tygerberg Hospital, Cape Town), were reviewed.

**Results:**

Median age at presentation from both countries was 66 years (interquartile range, IQR 61–73 years) while the median PSA at presentation was 46 ng/ml (IQR 16–336.5 ng/ml). Half of the men (117/234) presented with locally advanced disease while metastatic disease was observed in 65.9% (27/41) of Nigerian men and 34.1% (14/41) of South African men. Thirty-three per cent of the men presented with organ-confined disease. Overall, Nigerian men presented with less organ-confined disease and significantly higher stage of disease (*p* < 0.001). Risk stratification using PSA, Gleason scores and T-staging showed that 84.2% (*n* = 197) of all the men presented with high-risk PCa disease. There was a statistically significant difference between Nigerian and South African black men (*p* = 0.003) in terms of disease risk at presentation. Logistic regression analysis showed that age (Adjusted OR 1.053 (95% CI 1.003–1.106), *p* = 0.003) and country of residence (Adjusted OR 4.281 (95% CI 1.690–10.844), *p* = 0.002) had a statistically significant relationship with high risk of PCa while disease co-morbidities (like diabetes and hypertension) and rural/urban location in both countries did not.

**Conclusions:**

Disparities exist between PCa presentation and clinicopathologic behaviour among Nigerian and South African black men. Nigerian men showed higher disease risk at presentation. Environmental-genetic interactions need further exploration in the aetio-pathogenesis of PCa in black men of African ancestry.

## Background

Prostate cancer (PCa) represents a foremost cancer among men and the commonest non-cutaneous malignancy globally in men [1]. It is a disease of the ageing man, with over 33% of men above the age of 60 years presenting with a latent form of PCa [2]; thus the disease is likely to be more prevalent than is currently documented. It is known to be particularly more aggressive among men of African descent who usually present with more advanced disease and have poorer clinical outcomes that are associated with higher mortality [3–5]. This has been the observed trend among black men resident within and outside Africa [6–12]. The high PCa burden in Africa has been attributed to delayed diagnosis and treatment, poor cancer awareness and low level of cancer screening programs. Some environmental and genetic factors have also been implicated [11, 12]. There is, however, a dearth of PCa research in men of African ancestry resident in Africa to unravel this continued trend among black men. Nigeria and South Africa, ranked number 1 and 3 on the continent in terms of gross domestic product (GDP), respectively, combined have the highest cumulative incidence of PCa in Africa [10].

Medical co-morbidities such as cardiovascular diseases like hypertension, type 2 diabetes and benign prostate hyperplasia (BPH) could impact on the presentation of prostate cancer and eventually determine its outcome during or after treatment [13]. Men with co-morbidities may be overtreated even when they have low risk disease, thus compromising their quality of life [14]. A Jamaican study showed that rural-dwelling black men presented with higher risk of PCa when compared with urban-dwelling black men [15]. Similarly, a Canadian study showed association between occupation, industry and PCa, with farming activities having an higher risk of PCa disease presentation, followed by being a member of the armed forces, legal services, office work and plumbing, and in addition, duration of employment of more than 10 years [16]. Being married is also considered a form of social support that was associated with localized disease, more definitive treatment and better disease survival [17, 18].

We therefore set out to look at the clinical and pathological characteristics of PCa (presenting prostate specific antigen (PSA) level, histopathological type, tumour grade and stage of disease) among black men of Nigeria and South Africa; with a view to compare the characteristics of the disease and its association with socio-demographic characteristics and medical co-morbidities among black men from both countries.

## Methods

This was a retrospective cross-sectional study in which we abstracted anonymized data from de-identified hospital records of eligible black patients with PCa managed at National Hospital, Abuja, Nigeria and Tygerberg Hospital, Cape Town, South Africa between 2007 and 2017 using data extraction sheets. The data from Nigeria was abstracted from patient notes in clinical folders while those from South Africa were data collected prospectively into a secured database. Ethical approval to undertake the study was granted by the National Hospital, Abuja, Nigeria and Stellenbosch University, South Africa (NHA/EC/047/2018 and S17/10/248, respectively).

We used consecutive sampling technique on all the eligible patients records till the projected sample size of 234 (117 from each country) was reached. As a result of the generally late or poor health-seeking behaviour among Africans, the number of patients presenting in the hospitals for diagnosis and treatment of PCa is small across Africa and due to this [6, 8], we noted that consecutive sampling will give a random sample that is representative of the population for this study. It was easier and gave an expedited approach to the data collection.

Our selection criteria were male patients with complete data, seen and managed in either hospitals, racially classified or self-identified as black, with confirmed histopathological PCa diagnosis, according to the International Society of Urologic Pathology (ISUP) guidelines of 2005 and 2014 [19, 20]. To ensure consistency in both centres and as an added quality control measures, de-identified pathological slides of 5% of the total sample size from each centre were swopped and reviewed by Pathologists from both centres. Both pathologists were blinded in their reports of diagnosis and grading. Six pathological slides were submitted for review from Tygerberg Hospital, South Africa to Nigeria while five were submitted for review from National Hospital, Abuja to South Africa. Data collection proceeded upon indication that there was > 80% concordance in the pathology reports emanating from both centres.

We collected data such as age at diagnosis, demography (marital status, occupation and suburb of residence), medical co-morbidities and presenting pathologies (PSA, histological variant, Gleason scores and disease stage especially the T-stage). We used the Union for International Cancer Control (UICC) 2009 Tumour -Node-Classification (TNM) 7 for our T-definitions [21] [UICC/TNM 7 (2009) was mostly in use in both centres during the data collection period for this study]. Where MRI T-staging was not available, we relied on other clinical findings (such as digital rectal examination (DRE) among others) available in the patients’ records, to evaluate clinical staging at presentation. We then identified staging presentations of the patients as organ confined (T1, T2 only), locally advanced (T3/T4 only/any T, N1) or metastatic diseases (any T, any N, M1) [21].

Using PSA value at presentation, the patients were classified into low, intermediate and high-risk PSA categories. We also looked at the Gleason scores (GS) pathology grading to identify well (GS = 2–6), moderately (GS = 7) and poorly differentiated diseases (GS = 8–10) [19, 20]. We then combined the PSA, GS and T-staging presentation, using an adaptation*^1^ of D’Amico’s cut-off criteria [22] to risk stratify the patients into high (PSA > 20 or GS = 8–10 or ≥ T2c/any T), intermediate (PSA = 10–20 or GS = 7 or T2b) and low-risk (PSA < 10, GS = 2–6 and T1–T2a) disease categories. We thereafter compared these disease categories between both countries. [For the purpose of binary regression modelling alone, we combined the low and intermediate risk categories as low risk group (reference category) because of the low numbers of these groups in our study versus the high-risk group.]

Using a standard multi-variate logistic regression analysis, we further identified if there was any association between high risk PCa and socio-demographic factors like age, self-reported cigarette smoking and alcohol history, rural/urban residence, medical co-morbidities (type 2 diabetes, hypertension and BPH specifically) as well as country of residence (with South Africa as reference category). The dependent variable was high risk prostate cancer while the independent variables included the socio-demographic characteristics, medical co-morbidities and country of residence. All P-values were two-sided, and significance was set at 95%.

Our data were analysed using STATA IC 15 software statistical package.

## Results

Data from 234 black male patients from both countries were analysed. Median age of disease presentation was 66 years (Interquartile Range, IQR 61–73 years). The social demographics and pathologic characteristics of the men are summarized in Table 1.

**Table 1:** Socio-demographic and pathologic characteristics

Variables	Country		Total *N* = 234 (%)	*P* values
	Nigeria	South Africa		
	*n* = 117 (%)	*n* = 117 (%)		
**Age (years)**	67.0 (61.50–73.77)^¥^	66.0 (60.81–72.40)^¥^		
**Marital status**				< 0.001
Married	117 (100.0)	76 (65.0)	193 (82.5)	
Single	0 (0.0)	27 (23.0)	27 (11.5)	
Others^#^	0 (0.0)	14 (12.0)	14 (6.0)	
Occupation*				< 0.001
Non-office/environmental contact	32 (27.3)	21 (18.3)	53 (22.8)	
Office/administrative/white collar	35 (30.0)	10 (8.7)	45 (19.4)	
Retired and unemployed	50 (42.7)	84 (73.0)	134 (57.8)	
**Urban/rural**				< 0.001
Rural	61 (52.1)	26 (22.2)	87 (37.2)	
Urban	56 (47.9)	91 (77.8)	147 (62.8)	
**Hypertension**				0.186
Yes	45 (38.5)	55 (47.0)	100 (42.7)	
No	72 (61.5)	62 (53.0)	134 (57.3)	
**Diabetes**				1.000
Yes	19 (16.2)	19 (16.2)	38 (16.2)	
No	98 (83.8)	98 (83.8)	196 (83.8)	
**BPH**				0.001
Yes	16 (13.7)	2 (1.7)	18 (7.7)	
No	101 (86.3)	115 (98.3)	216 (92.3)	
**Smoking history**				0.001
Yes	14 (12.0)	34 (29.0)	48 (20.5)	
No	103 (88.0	83 (71.0)	186 (79.5)	
**Alcohol**				0.549
Yes	28 (24.0)	32 (27.4)	60 (25.6)	
No	89 (76.0)	85 (72.6)	174 (74.4)	
**PSA (ng/ml)**	52.8 (18.65–298.15) ^¥^	47.8 (11.36–492.40) ^¥^		
**Histopathology varian**t				0.156
Adenocarcinoma	117 (100.0)	115 (98.3)	232 (99.1)	
Squamous cell carcinoma	0 (0.0)	2 (1.7)	2 (0.9)	
**Gleason score**				0.002
2–6	12 (10.3)	25 (21.4)	37 (15.8)	
7	19 (16.2)	17 (14.5)	36 (15.4)	
8–10	86 (73.5)	75 (64.1)	161 (68.8)	
**T-staging**				< 0.001
T_x_, T_1_–T_2a_	15 (12.8)	44 (37.6)	59 (25.2)	
T_2b_	0 (0.0)	5 (4.3)	5 (2.1)	
T_2c_–T_4_	102 (87.2)	68 (58.1)	170 (72.7)	
**M-staging [distant metastases only (M**_**1b-c**_**)]**				<0.001
	27 (65.9)	14 (34.1)	41 (100)	

^¥^Median (interquartile range)

*No job records for two South Africans

^#^Widowed or divorced

Adenocarcinoma of the prostate gland was the commonest histological variant with 99.1% (232/234) of the men presenting with such in both countries (117 Nigerians and 115 South Africans). Only 0.9% (2/234) of the men (who were South Africans) presented with Squamous Cell Carcinoma histological variant in the available records reviewed in this study period. (We note as a limitation that the diagnosis of Squamous Cell Carcinoma was not verified histologically).

Median PSA at presentation for the entire cohort was 46 ng/ml (IQR 16–336.5 ng/ml). Maximum PSA recorded was 9890 ng/ml, while the minimum recorded was 1.3 ng/ml. Men were classified into high risk (PSA > 20 ng/ml), moderate risk (PSA = 10-20 ng/ml) and low risk (PSA < 10 ng/ml) categories based on PSA at presentation (Table 2). There was no statistically significant difference (*χ*^2^ = 5.43, *p* = 0.066) in PSA categories between the men in the entire cohort.

**Table 2:** PSA category

PSA category	Country		Total *n* = 234 *n* (%)
	Nigeria *n* = 117 *n* (%)	South Africa *n* = 117 *n* (%)	
High risk	86 (73.5)	75 (64.1)	161 (68.8)
Moderate risk	19 (16.2)	17 (14.5)	36 (15.4)
Low risk	12 (10.3)	25 (21.4)	37 (15.8)

*χ*^2^ = 5.43(degree of freedom, *df* = 2), *p* = 0.066

Half of all the men (117/234) presented with locally advanced stage of clinical disease; 61.5% (72/117) of these were Nigerians and 38.5% were South Africans. Forty-one men (41/234) presented with metastatic disease from both countries; of which 65.9% (27/41) were Nigerians and 34.1% (14/41) were South Africans. Seventy-six men (76/234) had organ-confined disease, out of which 23.7% (18/76) were Nigerians and 76.3% (58/76) were South Africans. There is a statistically significant difference (*p* < 0.001) between staging presentations in both countries with the Nigerian men presenting with higher stage of clinical disease (Table 3).

**Table 3:** Staging presentation

Staging	Country		Total *N* = 234 *n* (%)
	Nigeria *n* = 117 *n* (%)	South Africa *n* = 117 *n* (%)	
Metastatic	27 (23.1)	14 (12.0)	41 (17.5)
Locally advanced	72 (61.5)	45 (38.5)	117 (50.0)
Organ-confined	18 (15.4)	58 (49.5)	76 (32.5)

*χ*^2^ = 31.4 (*df* = 2), p < 0.001

Using GS, 105 men from both countries had poorly differentiated disease (GS = 8–10) with 52.4% (55/105) being Nigerians and 47.6% (50/105) South Africans. Eighty-eight men presented with moderately differentiated disease (GS = 7) with 58% (51/88) being Nigerians and 42% (37/88) South Africans while 36 men presented with well differentiated disease (GS = 2–6). Of the well-differentiated diseases, 30.6% (11/36) were Nigerians and 69.4% (25/36) were South Africans. Five South Africans had no record of GS grading.

Disease risk stratification combining T-staging, PSA and GS showed that 197 men presented with high-risk disease category (PSA > 20 or GS = 8–10 or ≥ T2c/any T) and this included 54.8% (108/197) Nigerians and 45.2% (89/197) South Africans. Twenty-two men had intermediate risk disease (PSA = 10–20 or GS = 7 or T2b) with 27.3% (6/22) being Nigerians and 72.7% (16/22) South Africans. Fifteen men had low-risk disease (PSA < 10, GS = 2–6 and T1–T2a) with 20% (3/15) being Nigerians and 80% (12/15) being South Africans. There exists a statistically significant difference between Nigerians and South African black men (*p* = 0.003) in terms of disease risk at presentation, with Nigerian men having higher disease risk category (Table 4).

**Table 4:** PCa disease risk classification (adaptation*^1^ of D’Amico’s risk criteria)

Disease category	Country		Total *N* = 234 *n* (%)
	Nigeria *n* = 117 *n* (%)	South Africa *n* = 117 *n* (%)	
High	108 (54.8)	89 (45.2)	197 (100.0)
Intermediate	6 (27.3)	16 (72.7)	22 (100.0)
Low	3 (20.0)	12 (80.0)	15 (100.0)

χ^2^ = 11.8 (*df* = 2), *p* = 0.003

A standard multi-variate logistic regression analysis was performed to assess the effect of age, smoking history, rural/urban location, medical co-morbidities (diabetes, hypertension and BPH) as well as country of residence on PCa risk category.

After adjusting for the other co-variates (Table 5), only age (Adjusted Odds Ratio, OR = 1.053 (95% Confidence Interval, CI 1.003–1.106), *p* = 0.003) and country of residence (Adjusted OR 4.281 (95% CI 1.690–10.844), *p* = 0.002) were shown to be statistically significant in the association with high risk of prostate cancer disease.

**Table 5:** Multivariable logistic regression

Variables	Crude OR (95% CI)	*P* value	Adjusted OR (95% CI)	*P* value
Age	1.051 (1.005–1.100)	0.030*	1.053 (1.003–1.106)	0.039*
Urban/rural	1.111 (0.533–2.314)	0.779	0.728 (0.313–1.696)	0.462
Smoking history	2.918 (1.364–6.241)	0.006	2.237 (0.987–5.070)	0.054
Hypertension	0.898 (0.440–1.835)	0.769	0.659 (0.291–1.491)	0.317
Diabetes	1.536 (0.641–3.680)	0.336	1.823 (0.666–4.986)	0.242
BPH	1.071 (0.294–3.899)	0.918	2.348 (0.532–10.367)	0.260
Country	3.775 (1.693–8.417)	0.001*	4.281 (1.690–10.844)	0.002*

*Significant at 95%

## Discussion

Prostate cancer is predominantly a disease of the ageing man. Among the strong risk factors associated with PCa, older age, family history and African ancestry have been identified [23, 24]. The median age of presentation of PCa from our study was 66 years (IQR 61–73 years), which is similar to the median age of presentation in several studies of PCa in black men across Africa [7, 9, 31] and globally [11, 25]. The black patients in the Surveillance, Epidemiology, and End Results (SEER) database also had a mean age of presentation at 64.7 years [26]. This is in contrast to a study done in Southern Nigeria which had the peak age of disease at 70–79 years [27] and another study in South Africa with a mean age of presentation of 71.6 years [28]. It is widely acknowledged that PSA screening has globally influenced the earlier age of PCa presentation in the last two decades [26]. However, since there are no large routine public PSA screening programmes across Africa, the earlier age of presentation of PCa in this study could possibly be attributed to the known aggressive nature of the disease in men of African roots. It is worthy of mentioning that we did not include family history in our data analysis even though it is a recognised strong risk factor for PCa. This was because of incomplete records of family history for majority of the patients from both countries.

The predominant histopathology variant in this study was Adenocarcinoma from both countries. Only 0.9% of the South African black men presented with squamous-cell carcinoma. This is not unexpected with the anatomy of the prostate gland. Similar predominant patterns of Adenocarcinoma variant were shown in studies from Trinidad and Tobago [29], Burkina Faso [9], Ghana [7], Nigeria [30], Senegal [31] and South Africa [32, 33]. A few studies [34, 35] reported the small cell variant and squamous cell histopathology but only as lesser variants.

Our study showed that most of the men from both countries presented with PSA > 20 ng/ml, indicating a higher risk category of disease at presentation. Head-to-head categorical analysis of both countries also showed that there was no statistically significant difference in the median PSA at presentation (*p* = 0.06) between black men from South Africa and Nigeria. This observation is in tandem with the earlier findings that black men of African ancestry usually present with higher PSAs consistent with more aggressive disease. It could also be implied that irrespective of country of residence, black men usually present in similar patterns of higher risk of disease category when analysing within the context of PSA alone.

The results of GS grading showed that only 15.4% of all the men had well-differentiated diseases (GS = 2–6) and of this South Africans were more. Most of the samples (44.9%) were poorly differentiated (GS = 8–10) and Nigerians had more poorly differentiated diseases. Sanchez-Ortiz et al. in their analysis of non-palpable PCa disease had similar findings of 43% of poorly differentiated disease versus 37% when African-American men were matched with White men in the US [36]. Magoha et al. noted in a study undertaken in Kenya [37] had a finding of 27% of poorly differentiated disease while Yarney et al. in Ghana [7] had similar finding of 27% of poorly differentiated disease in their studies. Our study could not show a statistically significant difference of poorly differentiated disease between the Nigerian and South African black men possibly because of our limited sample size, however the finding of Nigerians having more poorly differentiated and smaller well-differentiated samples is clinically significant. This finding may be consequent of the inherent tumour biology differences between the two black populations. To a lesser extent, it is also possible that the cancer awareness and advocacy has taken more roots in South Africa when compared to Nigeria thus somewhat influencing the earlier presentation. It could also be argued that even though both countries have somewhat similar public health systems, South Africa’s more robust when compared to Nigeria’s [38].

The staging presentation in our study was a combination of imaging and clinical records found in patients notes. It was noted that the South African records were more complete with respect to T-stage category of disease when compared to Nigeria. This might be due to more widespread use of imaging modalities [39] such as transrectal ultrasound (TRUS) and recently pelvic Magnetic Resonance Imaging (MRI) investigations in South Africa as compared to Nigeria. The fact that 50% of all the patients presented with advanced form of disease could lend credence to the inherent aggressive biologic nature of PCa among black men. It could also imply that in both countries, PCa presentation among black men is usually at a later stage as previously shown by various studies in Ghana [7], Burkina Faso [9], South Africa [40], Nigeria [30] and Senegal [31]. Our study further goes to show that there was a statistically significant difference (*p* < 0.001) in staging presentation in both countries, with the Nigerian men presenting with a higher stage of disease. It could be that there is poor awareness of PCa in Nigeria when compared to South Africa, leading to late presentation of disease [41]. Moreover, there may be some inherent, yet to be identified, strong biological factors [42–44] that are responsible for the more advanced presentation of disease among Nigerians. Further studies are necessary in unravelling the environmental-genetic risk relationship of PCa as revealed by this study’s findings.

When the men in our studies were further risk stratified into low, intermediate and high risk PCa disease category using an adaptation of D’Amico’s criteria [22] by combining PSA values, GS and T-staging, there was a similar pattern with the finding above showing that 84% of all the men had high risk disease at presentation. There was a statistically significant difference (*p* = 0.003) between Nigerian and South African black men in terms of disease risk presentation, buttressing the earlier finding that PCa seems to be more aggressive among the Nigerian group. This was further corroborated with our regression analysis when we tried to establish the relationship between high risk PCa disease at presentation and socio-demographic factors like age, urban/rural location, smoking history, hypertension, type 2 diabetes mellitus, BPH and country of origin. Only age (Adjusted OR 1.053 (95% CI 1.003–1.106), *p* = 0.003) and country of residence (Adjusted OR 4.281 (95% CI 1.690–10.844), *p* = 0.002) were found to be statistically significant, after adjusting for other co-variates. Our findings indicated that being in Nigeria had four times the risk of presenting with the high risk PCa disease as against South Africa. It should be noted that the choice of these disease co-morbidities in our analysis was not because of any special relationship with PCa. However, these were the most co-morbidities reported in the patient records available to us in this study and could also impact on the timing of presentation of these patients in seeking the diagnosis and treatment of PCa [45, 46].

It is unknown why the Nigerian group were more at risk of presenting with high-risk disease, but we speculate that there might be genetic and/or environmental factors that influence tumour pathology. Fernandez and Zeigler-Johnson et al. [47] have shown that the frequency of risk alleles in Cytochrome P450 genes (CYP3A4 and CYP3A5) and Steroid 5-alpha reductase (SRD5A2) gene differ significantly between West and South African men. Moreover, Kumar et al. showed that unique West African dietary and social practices may additionally influence tumour progression [48].

Our study has several limitations which must be acknowledged as these might have impacted in the results we obtained. There were incomplete data for most patients from both countries. For example, only two South Africans in the entire cohort had lymph nodal status report, others were either not available or could not be assessed from the records. There is also the possibility of under or over-representing any of the population samples due to the consecutive sampling technique which we employed. This may have introduced selection bias into the study. It is also possible that our number of high-risk patients might have been inflated due to the addition of the metastatic patients to the high-risk category when we adapted the D’Amico’s criteria to our disease risk categorization.

## Conclusions

The aggressive nature of PCa among black men, irrespective of place of residence, is incontestable globally [2, 3, 5, 11, 12, 40]. This study has further demonstrated this in the clinical and pathological presentations of the black men from South Africa and Nigeria. The men from Nigeria tend to present with high-risk disease of PCa when compared with the black men from South Africa. This might imply that environmental-genetic interplay may also have a role to play in the aetio-pathogenesis of PCa and future studies need to explore more of this in the African setting

## Data Availability

The datasets used in this study are available from the corresponding authors on reasonable request. The datasets are also available in FIGSHARE data repository accessible using the weblink: https://figshare.com/s/890d18db9bdd6cd8a5c4.

## Abbreviations

BPH: Benign prostate hyperplasia
CI: Confidence interval
CYP3A4 and CYP3A5: Cytochrome P450 genes
DRE: Digital rectal examination
GDP: Gross domestic product
GS: Gleason score
ISUP: International Society of Urologic Pathology
IQR: Interquartile range
MRI: Magnetic resonance imaging
OR: Odds ratio
PCa: Prostate cancer
PSA: Prostate-specific antigen
SEER: Surveillance epidemiology and end results
SRD5A2: Steroid 5-alpha reductase gene
TNM: Tumour-node-classification
TRUS: Transrectal ultrasound
UICC: Union for International Cancer Control (UICC)

## Footnotes

1 While it is true that D'Amico's criteria are only applicable in localized diseases in clinic setting, we have adapted the cut-off points to include metastatic patients in our study for purposes of epidemiological/statistical comparisons as these align with the objectives of this study. There is no clinical significance behind this.
